# Practical Treatise on Surgical Diagnosis

**Published:** 1880-12

**Authors:** 


PRACTICAL TREATISE ON SURGICAL DIAGNOSIS. Designed as a Manual for Practitioners and Students. By Ambrose L. Ranney. A M., M.D., Adjunct Professor of Anatomy, and late Lecturer on the Surgical Diseases ot the Genito-Urinary Organs, and on Minor Surgery in the Medical Department of the University of the City of New York, etc., etc. Second edition. Enlarged and revised. Wm. Wood & Co., N. Y. 1880.

This is strictly a book for diagnosis, and the author has attempted, in presenting questions of etiology, pathology and treatment, to discuss them as briefly as the nature of the case would allow. Commencing with diseases of the blood vessels, each disease of this system is detailed and compared, when the next, diseases of the joints, is gone through with in the same manner. A chapter each is devoted to diseases of bone, dislocations, fractures, diseases of the male genitals, diseases of the abdominal cavity and diseases of tissues. As a ready reference book to the practitioner, and as a text book to the student, we commend the work.

